# Estimation of CO_2_ Separation Performances through CHA-Type Zeolite Membranes Using Molecular Simulation

**DOI:** 10.3390/membranes13010060

**Published:** 2023-01-03

**Authors:** Yasuhisa Hasegawa, Mayumi Natsui, Chie Abe, Ayumi Ikeda, Sean-Thomas B. Lundin

**Affiliations:** National Institute of Advanced Industrial Science and Technology (AIST), Research Institute for Chemical Process Technology, Sendai 983-8551, Japan

**Keywords:** CO_2_ separation, zeolite membrane, chabazite, grand canonical Monte Carlo, molecular dynamics, Maxwell–Stefan equation

## Abstract

Chabazite (CHA)-type zeolite membranes are a potential material for CO_2_ separations because of their small pore aperture, large pore volume, and low aluminum content. In this study, the permeation and separation properties were evaluated using a molecular simulation technique with a focus on improving the CO_2_ separation performance. The adsorption isotherms of CO_2_ and CH_4_ on CHA-type zeolite with Si/Al = 18.2 were predicted by grand canonical Monte Carlo, and the diffusivities in zeolite micropores were simulated by molecular dynamics. The CO_2_ separation performance of the CHA-type zeolite membrane was estimated by a Maxwell–Stefan equation, accounting for mass transfer through the support tube. The results indicated that the permeances of CO_2_ and CH_4_ were influenced mainly by the porosity of the support, with the CO_2_ permeance reduced due to preferential adsorption with increasing pressure drop. In contrast, it was important for estimation of the CH_4_ permeance to predict the amounts of adsorbed CH_4_. Using molecular simulation and the Maxwell–Stefan equation is shown to be a useful technique for estimating the permeation properties of zeolite membranes, although some problems such as predicting accurate adsorption terms remain.

## 1. Introduction

Zeolite membranes separate via molecular sieving and selective adsorption, which makes them a promising candidate technology for energy-efficient separations. Geus et al. successfully formed polycrystalline MFI-type zeolite layer on a porous substrate and investigated the permeation properties of hydrocarbons [1,2,3,4,5]. In the 1990s, Kita and coworkers developed a commercially available LTA-type zeolite membrane and applied it to dehydration of ethanol [6,7,8].

Although it is well known that polymeric membranes are currently employed [9,10,11,12], zeolite membranes exhibit excellent dehydration performance. FAU, DDR, AEI, and CHA-type zeolite membranes show high CO_2_ separation performance [13,14,15,16,17,18,19,20,21,22,23,24,25,26,27,28]. Kusakabe et al. developed the FAU-type zeolite membranes, and the influence of the membrane composition and cations species on the adsorption and diffusion properties of CO_2_ in the zeolite were investigated to improve the CO_2_ separation performance [13,14,15,16]. As a result, the CO_2_ permeance and CO_2_/N_2_ permeance ratio were ca. 10^−6^ mol m^−2^ s^−1^ Pa^−1^ and 40–100, respectively. Noble and coworkers investigated a SAPO-34 membrane and applied it to CO_2_ separation from CH_4_ [17,18,19]. The CO_2_ permeance and CO_2_/CH_4_ permeance ratio were ca. 10^−6^ mol m^−2^ s^−1^ Pa^−1^ and 80–170, respectively. DDR-type zeolite membranes [20,21] exhibited a higher CO_2_/CH_4_ permeance ratio of 200–2000, although the CO_2_ permeance was lower than those of FAU and CHA-type zeolite membranes. Recently, we have developed a high-silica CHA-type zeolite membrane (Si/Al = 18) for CO_2_ separation [26,27,28]. The CO_2_ permeance and CO_2_/CH_4_ permeance ratio were ca. 5 × 10^−7^ mol m^−2^ s^−1^ Pa^−1^ and 200, respectively. This is important for improving the CO_2_ separation performance to understand the permeation mechanisms in zeolite membranes.

There are many reports about the permeation and separation mechanisms in zeolite membranes [3,4,5,14,15,16]. Since CO_2_ and hydrocarbon molecules adsorb onto zeolite strongly, the adsorbed molecules move to a neighboring adsorption site according to the concentration gradient across the membrane. The permeation phenomenon due to the surface diffusion is quantitatively described by the Maxwell–Stefan equation [3,4,5]. Many studies have predicted the permeation and separation properties of zeolite membranes from the adsorption and diffusion properties of single gases. Bakker et al. checked that the equation is suitable for expression of the single gas permeation properties through a silicalite-1 membrane [4]. Additionally, van den Broeke et al. applied the equation to separation of binary hydrocarbon mixtures [5], and the gas permeation properties for binary mixtures could be described using the adsorption and diffusion parameters obtained by single component gases.

The permeation and separation properties of zeolite membranes are explained by adsorption of molecules on zeolite and diffusion in zeolite channels. Both the adsorption and diffusion properties can be predicted by molecular simulation such as grand canonical Monte Carlo and molecular dynamics, respectively [29,30,31,32,33,34]. Vujic et al. reported the potential parameters applicable to many zeolites [33].

In this study, the CO_2_ separation performances of CHA-type zeolite membranes with Si/Al = 18 were predicted using the molecular simulation technique and Maxwell–Stefan equation to understand the permeation behavior in CHA-type zeolite membranes.

## 2. Theory

### 2.1. Molecular Simulation

The interaction between adsorbate and adsorbent atoms is described as the sum of interactions between bonded and nonbonded atoms as [33]:

(1) $$\Phi_{t}=\Phi_{\mathrm{bond}}+\Phi_{\mathrm{non}-\mathrm{bond}}.$$

The interaction between bonded atoms is calculated as the sum of bond-stretching and angle-bending as:

(2) $$\Phi_{\mathrm{bond}}=\Phi_{\mathrm{bond}-\mathrm{stretch}}+\Phi_{\mathrm{angle}-\mathrm{bend}},$$

(3) $$\Phi_{\mathrm{bond}-\mathrm{strech}}=\frac{1}{2}k_{b}{\left(r-r_{0}\right)}^{2},$$

(4) $$\Phi_{\mathrm{angle}-\mathrm{bend}}=\frac{1}{2}k_{\theta }{\left(\theta -\theta_{0}\right)}^{2},$$

where *k*_b_ and *k*_θ_ are the force constants for bond-stretching and angle-bending, respectively. The interaction between nonbonded atom pair is calculated as the sum of van der Waals and coulomb interactions as:

(5) $$\Phi_{\mathrm{non}-\mathrm{bond}}=\Phi_{\mathrm{vdW}}+\Phi_{\mathrm{coulomb}},$$

(6) $$\Phi_{\mathrm{vdW}}=4\phi_{ij}\left[{\left(\frac{\sigma_{ij}}{r_{ij}}\right)}^{12}-{\left(\frac{\sigma_{ij}}{r_{ij}}\right)}^{6}\right],$$

(7) $$\Phi_{\mathrm{coulomb}}=\frac{1}{4\pi \varepsilon_{0}}\cdot \frac{Q_{i}Q_{j}}{r_{ij}},$$

where the depth of interaction *ϕ_ij_* and zero-interaction distance *σ_ij_* for the pair of different atoms are calculated as:

(8) $$\phi_{ij}=\sqrt{\phi_{i}\phi_{j}},$$

(9) $$\sigma_{ij}=\frac{1}{2}(\sigma_{i}+\sigma_{j}).$$

When two atoms are in the same structure and separated by three covalent bonds (known as a 1–4 interaction), the interaction is treated as a nonbonded interaction with scaling factor of 0.5. Nonbonded interactions are ignored for directly bonded atoms (1–2 interaction) and two atoms separated by two bonds (1–3 interaction) since they are included in the bond-stretching and angle-bending interactions.

### 2.2. Gas permeation through Zeolite Layer

**Figure 1:**
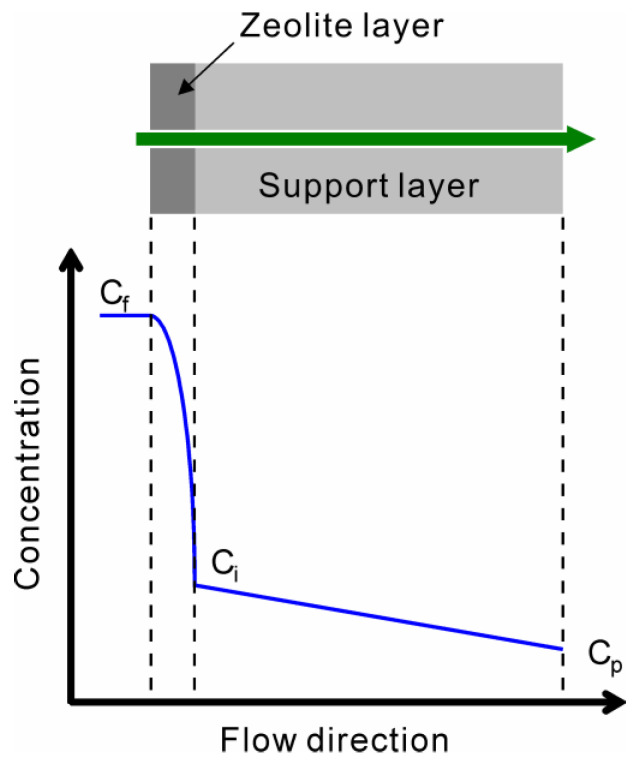
Schematic illustration of concentration gradient across the zeolite membrane supported by porous substrate. C_f_ is the concentration in the feed, C_p_ is the concentration in the permeate, and C_i_ is the concentration at the interface of the zeolite and support layers.

Zeolite membranes are often prepared on porous supports, as shown in Figure 1. The molecules are transferred by the concentration gradient across the membrane.

In the zeolite layer, molecules adsorbed on the adsorption sites within zeolite channels, and then move to a neighboring site according to the concentration gradient. The permeation flux is described as [3,4,5]:

(10) $$\left(\begin{array}{c} J_{1}\\ \vdots \\ J_{n} \end{array}\right)=-\varepsilon \rho \left[\begin{array}{ccc} a_{1} & \cdots & 0\\ \vdots & \ddots & \vdots \\ 0 & \cdots & a_{n} \end{array}\right]{\left[\begin{array}{ccc} B_{11} & \cdots & B_{1n}\\ \vdots & \ddots & \vdots \\ B_{n1} & \cdots & B_{nn} \end{array}\right]}^{-1}\left[\begin{array}{ccc} \Gamma_{11} & \cdots & \Gamma_{1n}\\ \vdots & \ddots & \vdots \\ \Gamma_{n1} & \cdots & \Gamma_{nn} \end{array}\right]\left(\begin{array}{c} \nabla \Theta_{1}\\ \vdots \\ \nabla \Theta_{n} \end{array}\right).$$

The elements of matrix *B* are calculated by:

(11) $$B_{ii}=\frac{1}{D_{i}}+\sum_{\begin{array}{l} j=1\\ j\neq i \end{array}}^{n}\frac{\Theta_{j}}{D_{ij}},\;B_{ij}=-\frac{\Theta_{i}}{D_{ij}},$$

where the mutual diffusivity can be approximated as:

(12) $$D_{ij}=D_{i}^{\Theta_{i}/(\Theta_{i}+\Theta_{j})}D_{i}^{\Theta_{j}/(\Theta_{i}+\Theta_{j})}.$$

When the adsorption isotherm is described by a Langmuir Equation (13), the elements of matrix Γ are described using Equation (14).

(13) $$q_{i}=a_{i}\Theta_{i}=\frac{a_{i}b_{i}p_{i}}{1+\sum b_{i}p_{i}},$$

(14) $$\Gamma_{ij}=\delta_{ij}+\frac{\Theta_{i}}{1-\sum_{i}^{n}\Theta_{i}},$$

where *δ_ij_* = 1 for *i* = *j*, and *δ_ij_* = 0 for *i* ≠ *j*. The adsorption and diffusion parameters are summarized in Tables 2 and 3, respectively.

### 2.3. Mass Transfer in the Support Tube

In the porous support tube, the overall permeation flux is:

(15) $$J_{t}=\sum_{i}^{n}J_{i}=D\frac{dC}{dL},$$

where *L* is the thickness of the support, and *C* is the concentration shown by:

(16) $$C=\frac{\varepsilon p}{RT},$$

where *ε* is the porosity of the support. The diffusivity in the porous support is estimated by the Fuller equation [4,35]:

(17) $$D=\frac{1.01\times 10^{-5}T^{1.75}{\left(M_{i}^{-1}+M_{j}^{-1}\right)}^{1/2}}{p{\left(V_{i}^{1/3}+V_{j}^{1/3}\right)}^{2}},$$

where *M_i_* is the molecular mass of component *i* and *V_i_* is the diffusion volume of component *i*. The diffusion volumes of CO_2_ and CH_4_ were taken as 26.9 cm^3^ and 25.1 cm^3^, respectively [35].

## 3. Methods

### 3.1. Adsorption on Zeolites

The adsorption isotherms of CO_2_ and CH_4_ on the CHA-type zeolite were simulated by a grand canonical Monte Carlo (GCMC) technique using software (Biovia, Materials Studio 2021 Sorption). For the GCMC simulation, fugacity was applied to the canonical ensemble, and the number and location of molecules with the lowest potential energy were calculated probabilistically. The cutoff distance of the van der Waals interaction was 1.25 nm, and the Ewald summation method was used for the integration of the coulomb interaction. The total number of Monte Carlo cycles were 10^6^, and the average of the final 10^5^ steps were used as the simulation result. The fugacity was assumed to be equal to the pressure in this study since the difference between fugacity and pressure is less than 5% below 1 MPa.

**Figure 2:**
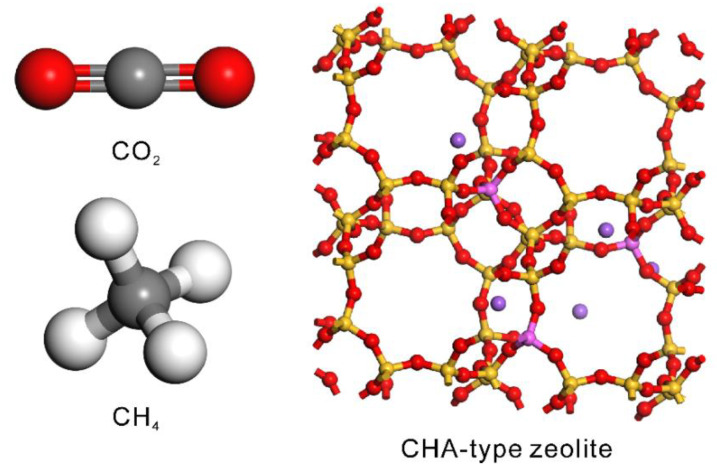
Atomistic models of CO_2_, CH_4_, and CHA-type zeolite used in this study.

Figure 2 shows the atomistic models of CO_2_, CH_4_, and CHA-type zeolite. The model of the CO_2_ molecule reported by Harris et al. [36] was used. This model can describe the gas–liquid coexistence curve including the critical point region. The carbon atom was connected to two oxygen atoms by chemical bonds 0.1149 nm long, and the bond-stretching was ignored (*k*_b_ = 0). The original angle of O=C=O was 180°, and the force constant was *k*_θ_ = 1236 kJ mol^−1^ rad^−2^. For CH_4_, the model reported by Siepman et al. [37] was used. The carbon atom was connected to four hydrogen atoms with bond lengths of 0.11 nm, and each H-C-H angle was 109.5°. Although the bond-stretching and angle-bending are ignored in this model (*k*_b_ = *k*_θ_ = 0), the gas–liquid coexistence curve can be expressed. The crystal structure of the CHA-type zeolite was imported from the IZA zeolite database [38]. The CHA-type zeolite model with a composition of Si_91_Al_5_Na_5_O_192_ was prepared by substituting Si atoms with Al atoms followed by introduction of Na^+^ cations by GCMC simulation. Table 1 lists the non-bonding interaction parameters for CO_2_, CH_4_, and zeolite. Vujic et al. reported that the adsorption of gases such as CO_2_ on CHA-type zeolite can be predicted with high accuracy by using these parameters [33].

### 3.2. Diffusion in Zeolite

The self-diffusivities of CH_4_ and CO_2_ in CHA-type zeolite channels were also simulated by a molecular dynamic technique (Biovia, Materials Studio 2021 Forcite Plus). CH_4_ and CO_2_ molecules were adsorbed at 1 MPa by GCMC, and the molecular dynamic simulation was conducted with a time step of 2 fs. The total simulation time was 1 ns, and the mean square displacement every 10 ps was plotted against the simulation time. The self-diffusivity was calculated using the slope by the Einstein equation. The procedure was repeated 5 times and the average value taken as the diffusivity.

## 4. Results and Discussion

### 4.1. Adsorption Isotherms

**Figure 3:**
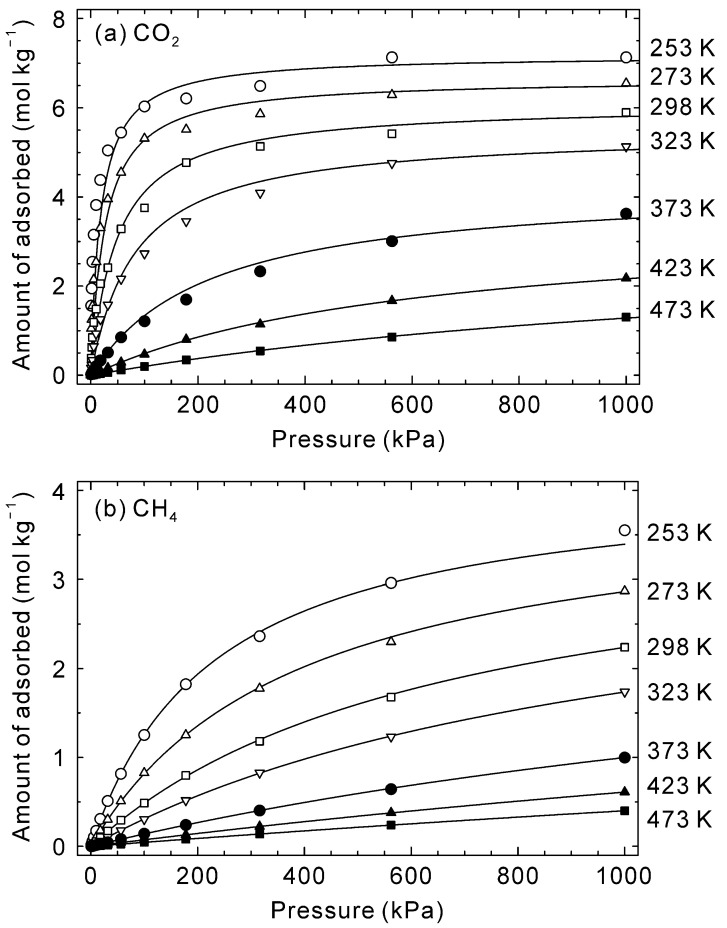
Simulated adsorption isotherms of (**a**) CO_2_ and (**b**) CH_4_ on CHA-type zeolite with Si/Al = 18.2 at 253–473 K. Symbols describe the simulated data, and lines are calculated values by Equation (13) and Table 2.

Figure 3 shows the adsorption isotherms of CO_2_ and CH_4_ on the CHA-type zeolite with Si/Al = 18.2 at 253–473 K. The amounts of adsorbed CO_2_ at 253 K increased significantly at low pressures and was 6.0 mol kg^−1^ at 100 kPa. At higher pressures, in contrast, the increment became small, with 7.1 mol kg^−1^ adsorbed at 1000 kPa. This isotherm is typical for adsorption in micropores, and the relationship is described by the Langmuir equation (Equation (13)). The adsorption isotherms of CO_2_ became linear as temperature increased. A similar trend was observed for CH_4_. The estimated isotherms were calculated for each temperature using the simulated points by Equation (13) and are shown as lines in Figure 3. The agreement between simulated points and estimated isotherm suggests the Langmuir equation is applicable at 253–473 K. Furthermore, the adsorption isotherms of CO_2_ and CH_4_ on CHA-type zeolite have been reported by several groups [39,40] and our simulated isotherms agree well with their experimental data, which suggests that the potential parameters are reasonable for simulating the adsorption and diffusion behaviors of CO_2_ and CH_4_ for CHA-type zeolite.

**Figure 4:**
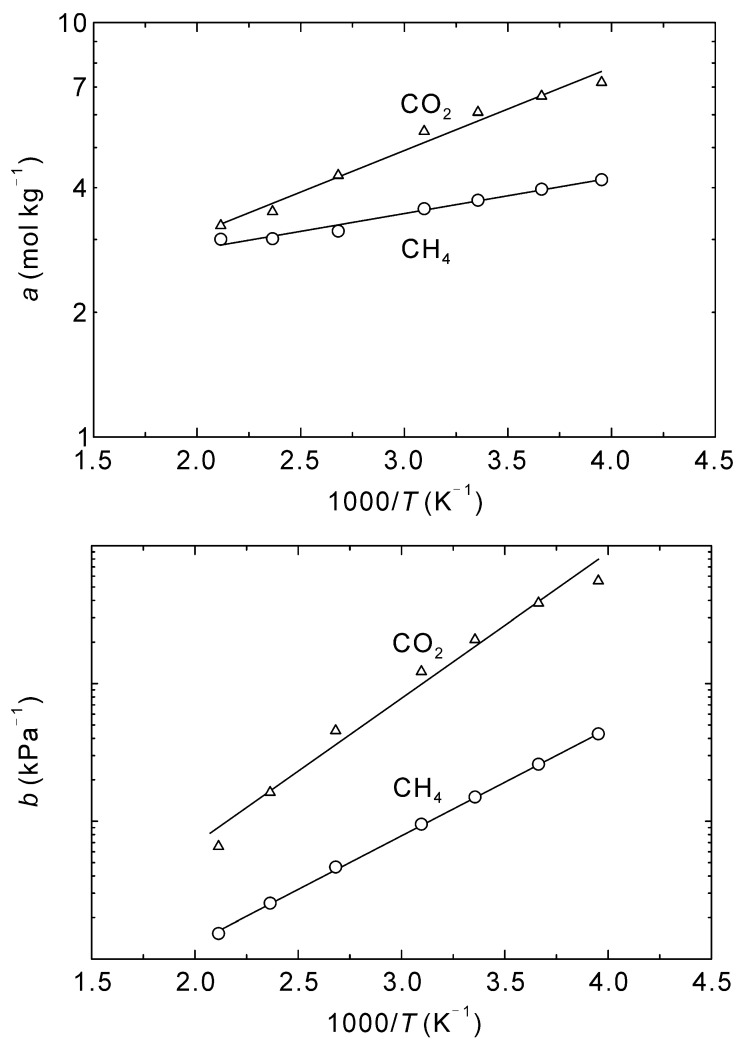
Arrhenius plots of amount of adsorbed at saturation *a* and Langmuir constant *b* for CO_2_ and CH_4_ for the adsorption on CHA-type zeolite with Si/Al = 18.2.

Figure 4 shows the effect of temperature on the adsorption amounts at saturation and Langmuir constants of CO_2_ and CH_4_. Both the saturated adsorption amounts, *a*, and Langmuir constants, *b*, decreased with increasing temperature. An Arrhenius dependence was observed, as is typical for adsorption isotherms, with the temperature dependencies described by:

(18) $$a_{i}=a_{i}^{*}\exp\left(-\frac{E_{a}}{RT}\right),\;$$

(19) $$b_{i}=b_{i}^{*}\exp\left(-\frac{E_{b}}{RT}\right).$$

The pre-exponential factors and activation energies are listed in Table 2. Assuming the heat of adsorption is equal to −(*E_a_* + *E_b_*), the heats of adsorption for CO_2_ and CH_4_ are 23.8 kJ mol^−1^ and 16.6 kJ mol^−1^, respectively. Maghsoudi et al. experimentally measured the heats of adsorption of CO_2_ and CH_4_ to be 21.0 kJ mol^−1^ and 17.1 kJ mol^−1^, respectively [40], which shows good agreement with the current work and further justifies the proposed methods for simulating the adsorption and diffusion behaviors of CO_2_ and CH_4_ in CHA-type zeolite.

### 4.2. Diffusivities

**Figure 5:**
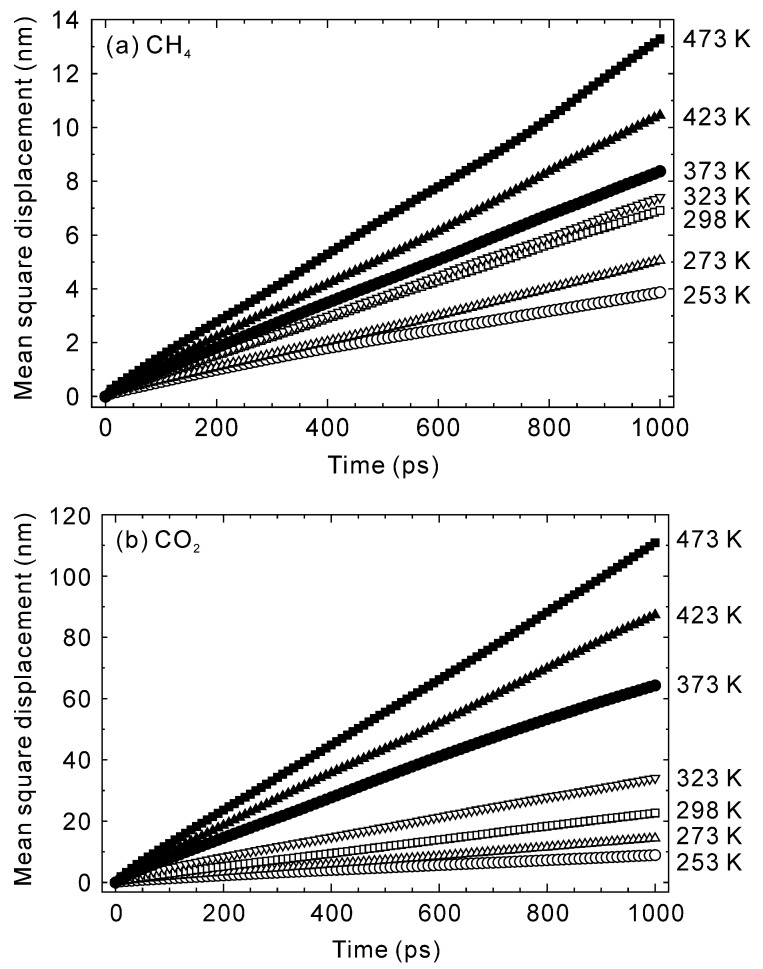
Time courses in the mean square displacements of (**a**) CH_4_ and (**b**) CO_2_ at 253–473 K.

**Figure 6:**
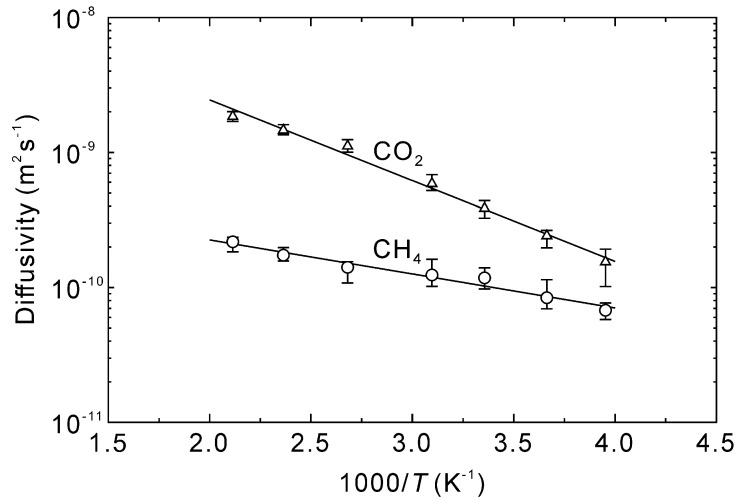
Arrhenius plots of CO_2_ and CH_4_ diffusivities within the CHA-type zeolite channels.

Figure 5 shows the time courses in the mean square displacement of CO_2_ and CH_4_ at 298–473 K. Because the mean square displacements were linearly proportional to simulation time before 1 ns, longer diffusional times were not required. The diffusivities of CO_2_ and CH_4_ in the CHA-type zeolite were calculated as 1/6 of the slope [33], which resulted in temperature dependencies as reported in Figure 6. The diffusivities of CO_2_ and CH_4_ at 298 K were 3.9 × 10^−10^ m^2^ s^−1^ and 1.2 × 10^−11^ m^2^ s^−1^, respectively. The pore diameter of the CHA-type zeolite is 0.38 nm [38], which is identical to the molecular diameter of CH_4_ (0.38 nm [41]). In contrast, the molecular diameter of CO_2_ (0.33 nm [41]) is smaller than the pore diameter, which results in molecular sieving behavior with a CO_2_ diffusivity nearly an order of magnitude higher than CH_4_. 

Vujic et al. compared to the simulated CO_2_ diffusivities with those obtained by experiments [33], and the simulated diffusivities were twice higher than the experimental values for high silica zeolites. Krishna et al. [42] also simulated the diffusivities of CO_2_ and CH_4_ in all-silica CHA-type zeolite at 300 K. When the fugacities of CO_2_ and CH_4_ were 1 MPa, their diffusivities were ca. 4 × 10^−10^ m^2^ s^−1^ for CO_2_ and 8 × 10^−11^ m^2^ s^−1^ for CH_4_. The similarity in diffusivity measurements with the current work suggests aluminum and sodium do not have a significant effect on gas diffusivity. This is considered reasonable because only one aluminum and sodium atom are incorporated per cavity for a Si/Al ratio of 18.2, as shown in Figure 2.

The effect of temperature on the diffusivity is also described by the Arrhenius equation as follows:

(20) $$D_{i}=D_{i}^{*}\exp\left(-\frac{E_{d}}{RT}\right).\;$$

The diffusivities at infinite temperature and activation energies of CO_2_ and CH_4_ are listed in Table 3. Sladek et al. [43] investigated the relationship between the diffusivity and heat of adsorption for physical and chemical adsorption species and concluded that the activation energy for diffusion was 0.45 times the heat of adsorption. This compares well with the current work wherein the activation energy of CO_2_ diffusivity is 0.48 times the heat of adsorption. 

### 4.3. CO_2_ Separation Performance

**Figure 7:**
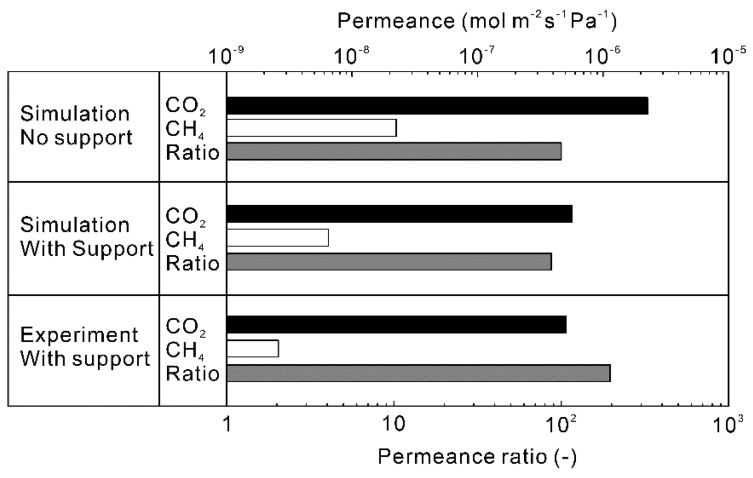
Comparison of simulated permeation properties of the CHA-type zeolite membrane with Si/Al = 18.2 to experimental results [28]. The permeation properties were calculated at CO_2_ concentration = 50 vol%, total pressure = 300 kPa, and temperature = 323 K. The thickness of zeolite layer was 5 μm, and the pore size, porosity and thickness of the support tube were 150 nm, 35% and 0.3 mm [24].

Figure 7 shows the influence of accounting the material transfer in porous support on the calculated permeation properties of CO_2_ and CH_4_ for the equimolar mixture at 323 K. When the polycrystalline zeolite layer was not supported by a substrate (self-standing membrane), the permeances of CO_2_ and CH_4_ were predicted to be 2.3 × 10^−6^ and 1.7 × 10^−8^ mol m^−2^ s^−1^ Pa^−1^, respectively. The permeances were reduced to 6.3 × 10^−7^ mol m^−2^ s^−1^ Pa^−1^ for CO_2_ and 5.0 × 10^−9^ mol m^−2^ s^−1^ Pa^−1^ for CH_4_ by supporting with the porous support (porosity = 35% and thickness = 0.3 mm). The reduction of CO_2_ permeance could be explained by the porosity of the support tube and pressure drop across the support (3.9 kPa). The calculated permeance of CO_2_ was almost identical to the experimental data [28]. However, the calculated CH_4_ permeance was higher than the experimental value. Since the amounts of adsorbed CO_2_ and CH_4_ were calculated using the extended Langmuir Equation (13) in this study, it is considered that the concentration gradient of CH_4_ across the polycrystalline zeolite layer was estimated to be high. As a result, the higher CH_4_ permeance was obtained compared to the experiment.

**Figure 8:**
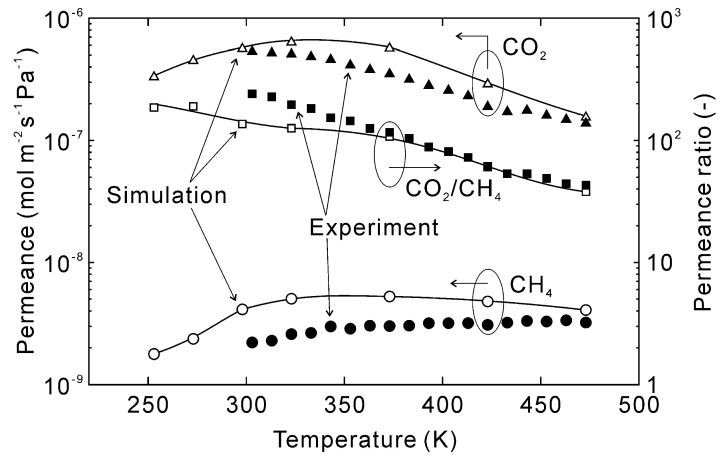
Effect of temperature on the permeation properties of the CHA-type zeolite membrane with Si/Al = 18.2. The permeation properties were calculated at CO_2_ concentration = 50 vol% and total pressure = 300 kPa, experimental data were taken from [28].

Figure 8 shows the effect of temperature on the estimated permeation properties of CO_2_ and CH_4_ for an equimolar mixture. The permeances of CO_2_ and CH_4_ at 253 K were 3.3 × 10^−7^ and 1.8 × 10^−9^ mol m^−2^ s^−1^ Pa^−1^, respectively, with a resultant CO_2_/CH_4_ permeance ratio of 190. The CO_2_ permeance increased with increasing temperature until reaching a maximum at 323 K and then decreasing with further rising temperatures. As a result, the CO_2_ permeance decreased to 1.5 × 10^−7^ mol m^−2^ s^−1^ Pa^−1^ at 473 K, with the permeance ratio also decreasing to 38. Notably, the simulated permeances followed similar trends as the experimental data and the permeances at 473 K were nearly identical. This convergence is because the effect of preferential adsorption was marginal at 473 K compared to lower temperatures, which suggests accurate prediction of permeation properties requires accurate estimates of adsorption amounts.

**Figure 9:**
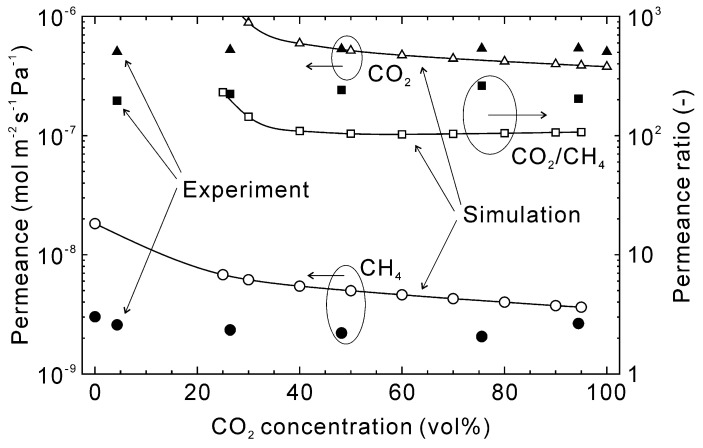
Influence of the CO_2_ concentration on the permeation properties of the CHA-type zeolite membrane with Si/Al = 18.2 at 303 K. The permeation properties were calculated at the total pressure of 300 kPa, and experimental data were taken from [28].

Figure 9 shows the influence of the CO_2_ concentration on the permeation properties of CO_2_ and CH_4_ at 303 K. The pure gas CO_2_ permeance was estimated to be 3.8 × 10^−7^ mol m^−2^ s^−1^ Pa^−1^ and slightly increased with decreasing CO_2_ concentration until around 40%. However, below 40% CO_2_ the CO_2_ permeance increased significantly. This was due to a relative change in the permeance versus partial pressure difference between feed and permeate streams. At 40% CO_2_, the partial pressures of CO_2_ on the feed and permeate sides were 120 kPa and 96 kPa, respectively, with a permeate flux of 1.5 × 10^−2^ mol m^−2^ s^−1^. At 30% CO_2_ concentration, the partial pressures and permeate flux were 90 kPa, 84 kPa, and 6.0 × 10^−3^ mol m^−2^ s^−1^, respectively. This means the relative permeate flux at 30% CO_2_ compared to 40% CO_2_ was 1/2.5, whereas the partial pressure difference was 1/4. As a result, the CO_2_ permeance increased below 40% CO_2_. For all conditions, the estimated CH_4_ permeance was higher than the experimental data, as discussed in Figure 7 and Figure 8, which is the cause of the lower CO_2_/CH_4_ permeance ratio.

**Figure 10:**
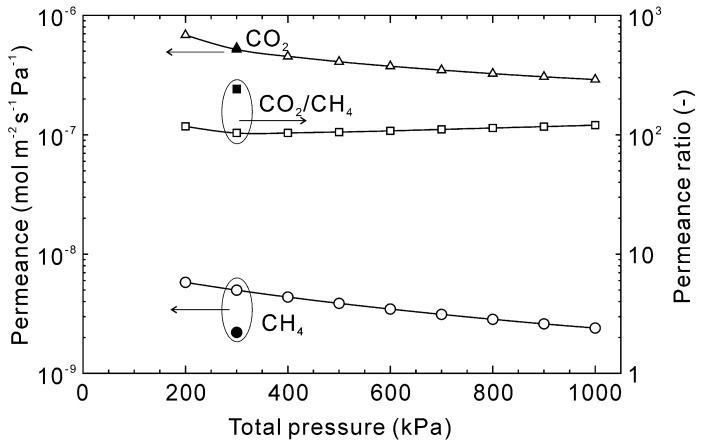
Influence of total pressure on the permeation properties through the CHA-type zeolite membrane with Si/Al = 18.2 at 303 K. The permeation properties were calculated at a CO_2_ concentration of 50 vol%, and experimental data were taken from [28].

Figure 10 shows the influence of the total pressure on the permeation properties of CO_2_ and CH_4_ at 303 K. When the total pressure was 200 kPa, the estimated permeances of CO_2_ and CH_4_ were 6.8 × 10^−7^ and 5.8 × 10^−9^ mol m^−2^ s^−1^ Pa^−1^, respectively, with a CO_2_/CH_4_ permeance ratio of 120. The permeances decreased with increasing total pressure and at 1000 kPa were 2.9 × 10^−7^ for CO_2_ and 2.4 × 10^−9^ mol m^−2^ s^−1^ Pa^−1^ for CH_4_. The decrease was calculated to be similar for both gases so the permeance ratio was nearly independent of total pressure.

## 5. Conclusions

In this study, the permeation and separation properties of a CHA-type zeolite membrane were evaluated for improving the CO_2_ separation performance. The adsorption isotherms of CO_2_ and CH_4_ on CHA-type zeolite with Si/Al = 18.2 were predicted by grand canonical Monte Carlo, and the diffusivities in zeolite micropores were simulated by molecular dynamics. The CO_2_ separation performance of the CHA-type zeolite membrane was estimated by a Maxwell–Stefan equation, accounting for mass transfer through the support tube. In this study, the influences of the support tube, temperature, CO_2_ concentration, and total pressure on the permeation properties were calculated, and the estimated permeation properties were compared with experimental data [24]. The estimated CO_2_ permeance agreed well with the experimental results due to the inclusion of the effect of the support tube. However, the estimated CH_4_ permeance was slightly overestimated, suggesting that better predictions of the amount of adsorbed CH_4_ on both the sides of the membrane must be made to obtain more accurate results. 

## Figures and Tables

**Table 1:** Nonbonding interaction parameters of CH_4_, CO_2_, and zeolite.

Molecule	Element	*σ* (nm)	*ε*/*k* (K)	*q* (e)	Ref.
CH_4_	C	0.3730	148.0	0	[33]
	H	---	---	0	
CO_2_	C	0.2757	28.1	0.6512	[32]
	O	0.3033	80.5	−0.3256	
Zeolite	Si	0.2970	32.0	1.413	[29]
	Al	0.3140	24.0	1.072	
	O(Si–O–Si)	0.3011	52.0	−0.7065	
	O(Si–O–Al)	0.3011	55.0	−0.8712	
	Na	0.3230	234.1	1.000	

**Table 2:** Pre-exponential factors and activation energies of CO_2_ and CH_4_ for the adsorption on CHA-type zeolite with Si/Al = 18.2.

	Unit	CO_2_	CH_4_
*a_i_* ^*^	mol kg^−1^	1.24	1.92
*E*_a_	kJ mol^−1^	3.8	1.6
*b_i_* ^*^	kPa^−1^	5.53 × 10^−6^	3.57 × 10^−6^
*E*_b_	kJ mol^−1^	20.0	15.0

**Table 3:** Diffusivities at infinite temperature and activation energies of CO_2_ and CH_4_ within CHA-type zeolite with Si/Al = 18.2.

	Unit	CO_2_	CH_4_
D_i_^*^	m^2^ s^−1^	3.8 × 10^−8^	7.2 × 10^−9^
*E_d_*	kJ mol^−1^	11.4	4.8

## Data Availability

Not applicable.

## Nomenclature

*a*	Amount adsorbed at saturation (mol kg^−1^)
*b*	Langmuir constant (Pa^−1^)
*B*	Mobility as defined by Equation (11)
*C*	Concentration (mol m^−3^)
*D*	Diffusivity (m^2^ s^−1^)
*E*	Activation energy (kJ mol^−1^)
*J*	Permeation flux (mol m^−2^ s^−1^ Pa^−1^)
*k*_b_	Force constant for bond-stretching (kJ mol^−1^ m^−2^)
*k*_θ_	Force constant for angle-bending (kJ mol^−1^ rad^−2^)
*L*	Thickness (m)
*M*	Molecular mass (g mol^−1^)
*p*	Partial pressure (Pa)
*Q*	Partial atomic charge (e)
*q*	Amount of adsorbed (mol kg^−1^)
*r*	Distance (m)
*R*	Gas constant (=8.314 J K^−1^ mol^−1^)
*T*	Temperature (K)
*V*	Diffusion volume (cm^3^)
*Symbols*	
Γ	Thermodynamic factor defined by Equation (14)
*δ*	Kronecker delta (dimensionless)
*ε*	Porosity (dimensionless)
*ε*_0_	Vacuum permittivity (=8.85 × 10^−12^ F m^−1^)
Φ	Interaction potential energy (kJ mol^−1^)
*ϕ*	Depth of potential (kJ mol^−1^)
*θ*	Binding angle (rad)
Θ	Surface coverage (dimensionless)
*ρ*	Density of zeolite (kg m^−3^)
*σ*	Distance at zero-potential energy (m)
